# Dual-emissive phenylalanine dehydrogenase-templated gold nanoclusters as a new highly sensitive label-free ratiometric fluorescent probe: heavy metal ions and thiols measurement with live-cell imaging†

**DOI:** 10.1039/d3ra03179a

**Published:** 2023-07-19

**Authors:** Mahsa Shahrashoob, Saman Hosseinkhani, Hanieh Jafary, Morteza Hosseini, Fatemeh Molaabasi

**Affiliations:** a Department of Biology, Science and Research Branch, Islamic Azad University Tehran Iran; b Department of Biochemistry, Faculty of Biological Sciences, Tarbiat Modares University Tehran Iran saman_h@modares.ac.ir; c Department of Life Science Engineering, Faculty of New Sciences & Technologies, University of Tehran Tehran Iran; d Department of Interdisciplinary Technologies, Breast Cancer Research Center, Biomaterials and Tissue Engineering Research Group, Motamed Cancer Institute, ACECR Tehran Iran

## Abstract

Phenylalanine dehydrogenase (PheDH) has been proposed as an ideal protein scaffold for the one-step and green synthesis of highly efficient multifunctional gold nanoclusters. The PheDH-stabilized fluorescent gold nanoclusters (PheDH-AuNCs) with dual emission/single excitation exhibited excellent and long-term stability, high water solubility, large Stokes shift and intense photoluminescence. Selectivity studies demonstrated that the red fluorescence emission intensity of PheDH-AuNCs was obviously decreased in less than 10 min by the addition of mercury, copper, cysteine or glutathione under the single excitation at 360 nm, without significant change in the blue emission of the PheDH-AuNCs. Therefore, the as-prepared PheDH-AuNCs as a new excellent fluorescent probe were successfully employed to develop a simple, rapid, low cost, label- and surface modification-free nanoplatform for the ultrasensitive and selective detection of Hg^2+^, Cu^2+^, Cys and GSH through a ratiometric fluorescence system with wide linear ranges and detection limits of 1.6, 2.4, 160 and 350 nM, respectively which were lower than previous reports. In addition, the results showed that PheDH-AuNCs can be used for the detection of toxic heavy metal ions and small biomarker thiols in biological and aqueous samples with acceptable recoveries. Interestingly, PheDH-AuNCs also displayed a promising potential for live-cell imaging due to their low toxicity and great chemical- and photo-stability.

## Abstract 2

PheDH as a new template is proposed for the synthesis of the AuNCs. The PheDH-AuNCs exhibit dual fluorescent emission with single excitation and can employed for the detection of Hg^2+^, Cu^2+^, Cys and GSH through a ratiometric fluorescence system.

## Graphical abstract

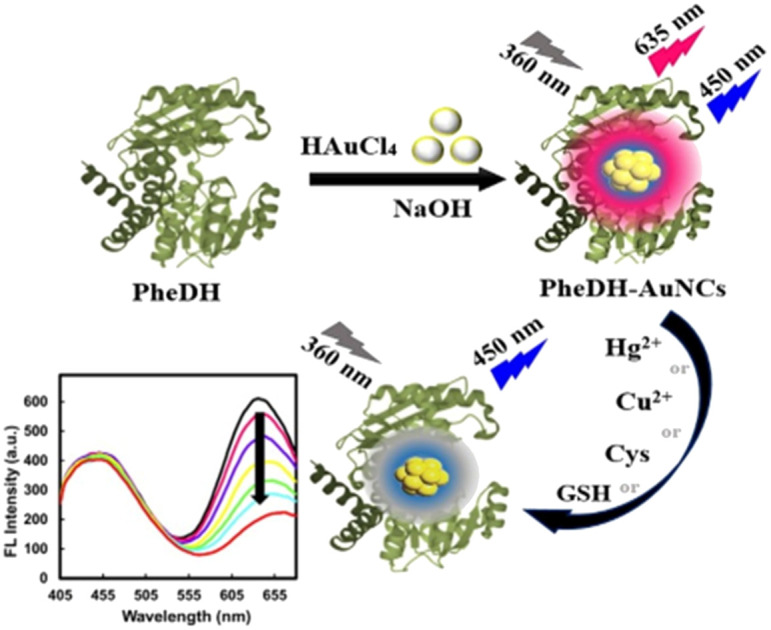

## 1. Introduction

The past decade has seen the rapid development of metal nanoclusters in many biological applications. Among the different nanoclusters, gold nanoclusters (AuNCs) have been considered as one of the most important nanomaterials due to their unique characteristics, such as excellent chemical and photo stability, strong fluorescent emission, facile preparation, low toxicity, good water solubility, catalytic activity and easy surface modification.^1–4^ In addition, AuNCs have great potential for biological and chemical sensing, bioimaging and therapy.^5,6^ Among suggested templates for the synthesis of AuNCs, protein-protected gold nanoclusters have received particular attention in nanobiotechnology because proteins have important functional groups (thiol, carboxyl and amine) and certain configurations that can be employed as appropriate capping/reducing agents for preparing fluorescent AuNCs.^7,8^ Protein-based AuNCs can exhibit environmental advantages, higher durability and enhanced luminescence. These platforms with the aim targeting can also be easily functionalized *via* protein coating layer on AuNCs.^9–11^ In addition, protein-protected AuNCs has been successfully employed as a fluorescent probe in sensing applications for the detection of various analytes (small molecules,^12,13^ metal ions,^14,15^ anion ions,^16^ drugs,^17^ proteases,^18^*etc.*) based on different mechanisms such as fluorescence quenching (signal off)^19^ or enhancement (signal on),^20^ fluorescence switching off–on^21^ and/or on–off,^22^ resonance energy transfer (FRET)^23^ and red^24^ or blue^25^ shift of emission peak.

The surface ligand is an important component in the formation of AuNCs as capping agent and plays a key role in enhancement of photoluminescence of nanoclusters by charge transfer mechanisms.^26–28^ Therefore, as the properties of the protein-stabilized AuNCs are highly influenced by the nature of protein templates, choosing an ideal protein scaffold is a great demand and important factor in the gold nanoclusters synthesis.^29,30^ Phenylalanine dehydrogenase (PheDH; EC 1.4.1.20) which is an important enzyme in food and pharmaceutical industries and medical diagnostic, belongs to a class of enzymes called oxidoreductases.^31,32^ PheDH catalyzes the reversible oxidative deamination of a broad range of hydrophobic amino acid substrates especially amino acids with aromatic side chains in the presence of oxidized nicotinamide adenine dinucleotide (NAD^+^) as a cofactor.^33,34^ This enzyme occurs in various microorganisms, but among these, *Bacillus badius* PheDH shows greater substrate specificity for l-phenylalanine.^35,36^ Recombinant histidine-tailed wild-type *Bacillus badius* PheDH has been selected for this study due to its suitable properties for the synthesis of AuNCs. This recombinant enzyme contains 13 tyrosine (Tyr), 6 cysteine (Cys) and 44 amine-containing residues and possibly other residues with reducing capability out of 380 amino acids in total. Since the key role of these amino acid residues in directing the formation of metal nanoclusters is elucidated,^37–39^ and this enzyme also has sufficient bulkiness for steric protection, so it can act as a great template to the preparing stable and label-free fluorescent AuNCs.

Recently, the ratiometric fluorescence sensing approach which provides two fluorescence emission peaks at different wavelengths under single excitation wavelength, has been proposed as an effective technique to overcome the limitations of the single emission sensing system.^40^ The main problem of these single emission-based sensors is that their fluorescence intensity can be affected by analyte concentration-independent factors such as light source fluctuations, photobleaching, variations in probe concentration, *etc.*, which results in inaccurate detection. In the ratiometric fluorescence system, the built-in self-calibration provided by calculating the intensity ratio of two fluorescence signals can minimize the influence of environmental factors and cause more accurate and sensitive detection.^41,42^ However, most of the previously reported ratiometric fluorescence sensors experience some practical disadvantages including interference between two fluorescence peaks, the need for external fluorophores, low biocompatibility, cytotoxicity, small Stokes shifts and the need for surface modification.^41,43^ It is interesting to note that only few researchers have reported ratiometric fluorescence assays using enzyme-protected nanoclusters.^43–45^

Among all the analytes, detection of heavy metals such as Hg^2+^ and Cu^2+^ have received increasing attention due to their high toxicity to the natural environment and human health.^46^ Detection of these ions in complicated environmental and biological samples may be difficult.^28^ Thus, developing a sensitive, selective and rapid sensing systems to detect them are essential. As well as these methods for detection of small biomarker thiols such as cysteine and glutathione (GSH) has attracted many interests, not only because of their vital roles in many biological processes, but also because these are important biomarkers for several diseases.^47,48^

**Scheme 1:**
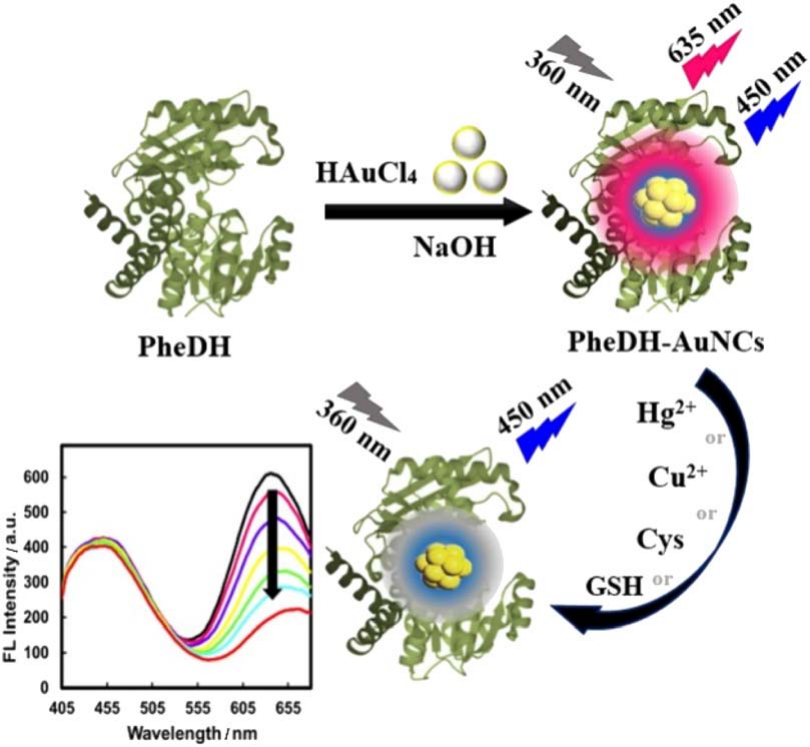
Schematic representation of the synthesis of PheDH-AuNCs and their application to Cu^2+^, Hg^2+^, Cys and GSH sensing *via* “ratiometric fluorescence” system.

In this study, we report for the first time the synthesis and characterization of multifunctional AuNCs using phenylalanine dehydrogenase enzyme as a biotemplate, under a one-step, simple, and “green” process. The resulting PheDH-stabilized AuNCs (PheDH-AuNCs) is highly stable, surface modification-free and biocompatible with dual fluorescence emissions under single excitation wavelength and large Stokes shift, which the emission peaks are located in two separate blue and red regions without any interference with each other. These features suggest that PheDH-AuNCs can offer a great potential for designing excellent label-free ratiometric sensing systems. Thus, we developed a selective, sensitive and facile method for the direct detection of mercury, copper, cysteine and glutathione targets using our PheDH-AuNCs as a fluorescence probe. The red fluorescence emission of PheDH-AuNCs can be effectively quenched in the presence of these targets while the blue emission of gold clusters exhibited no significant change (Scheme 1). On the other hand, the prepared PheDH-AuNCs can act as a great probe for the sensing of very low levels of these analytes in biological and aqueous samples. Furthermore, the excellent properties of PheDH-AuNCs made us eager to investigate the possibility of bioimaging. The findings indicated that the PheDH-AuNCs not only can sense toxic heavy metal ions and biomarker thiols but also can be successfully utilized for live cell imaging due to their low cytotoxicity, excellent photo/chemical stability and good cell permeability.

## 2. Experimental

### 2.1. Materials and reagents

Histidine-tailed wild-type *Bacillus badius* PheDH in *E. coli* BL21 containing the expression plasmid pET28a from Nora Gene Pishro Company (Iran), isopropyl-d thiogalactopyranoside (IPTG) and kanamycin from Sigma-Aldrich (USA), Ni-NTA Sepharose column affinity from Novagene, hydrogen tetrachloroaurate(iii) trihydrate (HAuCl_4_) from Alfa Aesar (USA) and NaOH, 3-(4,5-dimethylthiazol-2-yl)-2,5diphenyltetrazolium bromide (MTT), all metal salts and amino acids from Merck (Germany) were provided. All other chemicals were of analytical grade and used without further purification.

### 2.2. Apparatus

Fluorescence spectra were recorded using a Carry eclipse fluorescence spectrometer (Varian company, Australia). The ultraviolet-visible (UV-vis) absorption spectra were obtained by UV-vis spectrophotometer WPA (Biochrom, UK). Transmission electron microscopy (TEM) image was collected by a Philips CM300 electron microscope. The X-ray photoelectron spectroscopy (XPS) measurements were performed with an ESCALab220I-XL spectrometer (VG company, UK). The energy dispersive X-ray (EDAX) studies were carried out using a Hitachi S-4160 instrument. The hydrodynamic diameter and zeta potential were measured using a Zetasizer nano ZS series dynamic light scattering (DLS) (Malvern, UK). Fluorescence images were obtained using an Olympus fluorescence microscope equipped with a blue filter. Circular dichroism (CD) spectra were recorded using a J-715 JASCO CD spectrometer (JASCO, Japan).

### 2.3. Expression and purification of phenylalanine dehydrogenase enzyme

In order to purify native histidine-tailed *B. badius* PheDH, *E. coli* BL21 containing the plasmid pET28a was grown in LB medium with 50 μg mL^−1^ kanamycin and incubated overnight at 37 °C, 180 rpm. Then, 1 mL of this seeding was transferred to 250 mL of fresh TB medium containing kanamycin and incubated at 37 °C with vigorous shaking. Subsequently, when OD600 reached ∼0.6–0.8, the promoter was induced by addition IPTG (final concentration 0.8 mM) and the incubation was continued for 16 h at 18 °C with 180 rpm shaking. Then, after the cells were collected by centrifugation (6000 rpm for 10 min at 4 °C), the bacterial pellet was resuspended in lysis buffer (50 mM Tris, 300 mM NaCl and 10 mM imidazole, pH 7.8), and sonicated on ice to disrupt the bacterial cells. Subsequently, cell debris was removed by centrifugation (15 000 rpm for 20 min, at 4 °C). Then, PheDH enzyme was purified by (Ni-NTA) Sepharose affinity chromatography column, as reported earlier.^32^ The resulting supernatant was applied to column and washed with a buffer containing 40 mM imidazole in 50 mM Tris and 300 mM NaCl (pH 7.8). The histidine-tagged *Bacillus badius* PheDH was finally eluted from the chromatography column by increasing the imidazole concentration to 250 mM. The enzyme was dialyzed in glycine/KCl/KOH buffer (100 mM, pH ∼ 10.5) at 4 °C, and then sucrose was added (final concentration 0.5 M) and stored at 4 °C (Fig. S1†). The enzyme purity was analyzed by sodium dodecyl sulfate polyacrylamide gel electrophoresis (SDS-PAGE 12%); and purified protein concentration was estimated by “Bradford” assay. In order to determine enzyme activity, a reaction mixture containing glycine/KCl/KOH buffer (100 mM, pH ∼ 10.5), 25 μL l-phenylalanine (10 mM), 25 μL NAD^+^ (25 mM) and enzyme was prepared in a total volume of 250 μL. The PheDH activity was assayed by monitoring the formation of NADH at 340 nm during 1 min after injecting enzyme to the reaction solution.^49^

### 2.4. Synthesis of PheDH-capped AuNCs

All glassware was cleaned with aqua regia (HNO_3_/HCl, 1 : 3) and rinsed with ethanol and deionized water prior to use. In a typical synthesis of fluorescent AuNCs, equal volumes of aqueous solution of HAuCl_4_ (1 mM) and dialyzed purified PheDH (4.5 mg mL^−1^) were mixed under vigorous stirring at 37 °C. After 2 minutes, the pH of the solution was adjusted around 12.5 by adding NaOH (1 M) and the reaction was allowed to proceed for 24 h. Then, the product was purified through centrifugation (10 000 rpm, 10 min, 4 °C) to remove any larger particles, and the pale yellow supernatant was collected and stored at 4 °C for further use.

### 2.5. Optimization of the synthesis procedure

The experimental conditions for synthesis of PheDH-AuNCs such as reaction time, temperature, pH value and HAuCl_4_ concentration were optimized in the presence of a constant concentration of PheDH. Briefly, the reactions were conducted for varying reaction time periods from 30 min to 6 days at constant concentration of PheDH (4.5 mg mL^−1^, 1 mL) and different concentrations of HAuCl_4_ (0.25, 0.5, 1, 1.5, 2 and 3.5 mM, 1 mL) under constant temperature and pH condition (37 °C, ∼12.5). To investigate the effect of temperature and pH on the formation of gold nanoclusters, HAuCl_4_ (1 mM) and PheDH (4.5 mg mL^−1^) reaction at different pHs (5, 7.5, 10.5 and 12.5) and various temperatures (4, 27, 37, 47 and 57 °C) was carried out for 24 h.

### 2.6. Characterization of PheDH-AuNCs

Several methods have been utilized to characterize the prepared PheDH-AuNCs. The emission spectra, at an excitation wavelength of 360 nm, were recorded at room temperature. The morphology of as-synthesized PheDH-AuNCs were analyzed using a transmission electron microscopy (TEM). Circular dichroism (CD) spectra of aqueous solutions of the native PheDH and PheDH-AuNCs were recorded in far-UV region of <250 nm. The X-ray photoelectron spectroscopy (XPS) analysis was performed to clarify the oxidation state of AuNCs capped by PheDH. The existence of elemental gold was confirmed by energy dispersive X-ray (EDAX) analysis. The UV-vis absorption spectra in the range of 200 to 800 nm, hydrodynamic diameter and zeta potential of PheDH-AuNCs were also measured. The stability of PheDH-AuNCs was checked separately by monitoring their fluorescence emission properties every 5 min for 1 h (*λ*_ex_ = 360 nm), in the presence of high salt concentrations (1 M NaCl) and a pH range of 5–10.

### 2.7. Ratiometric assay

To investigate the selectivity of PheDH-AuNCs, the interference effects of different cations (Li^+^, Na^+^, K^+^, Ca^2+^, Sr^2+^, Ba^2+^, Al^3+^, Pb^2+^, Zr^4+^, Cr^3+^, Mn^2+^, Fe^3+^, Fe^2+^, Cu^2+^, Hg^2+^, Zn^2+^, Cd^2+^ in their nitrate or chloride forms) at final concentrations of 12 μM were examined by monitoring the emission spectra of the designed sensing system at an excitation wavelength of 360 nm under the same experimental conditions. Moreover, the fluorescence spectra of PheDH-AuNCs were recorded in the presence of various amino acids (Gly, Ala, Val, Ile, Met, Trp, Phe, Tyr, Ser, Thr, Cys, Gln, Arg, His, Glu, Asp) and GSH at final concentrations of 100 μM, after 7 min incubation.

Then, after observing the fluorescence quenching in the presence of Cu^2+^, Hg^2+^, Cys and GSH, the calibration curves were recorded by different concentrations of these analytes, separately. In a typical experiment, the prepared PheDH-AuNCs was diluted 12 times with deionized water and then titrated by additions of the target solution to give a final concentration of 209 nM for Hg^2+^, 245 nM for Cu^2+^ and 30 μM for Cys and GSH. The fluorescence spectra were subsequently recorded after the addition of each portion of the target solution (*λ*_ex_ = 360 nm). The fluorescence spectra were recorded for Hg^2+^ and Cu^2+^ (without incubation time) and also 10 min and 5 min after Cys and GSH addition, respectively.

### 2.8. Real samples analysis

The PheDH-AuNCs were used to detect Hg^2+^, Cu^2+^, Cys and GSH in real samples by applying the standard addition method. Copper and mercury solutions at final concentrations of 0 to 100 nM were separately spiked into the non-filtered tap and mineral water samples (3-fold dilution with deionized water) and the fluorescence spectra of PheDH-AuNCs were subsequently measured. For Cys and GSH detection in biological fluids, the fresh blood serum samples from healthy volunteers (with informed consent) were supplied by a local clinical laboratory in Tehran, and then the fluorescence spectra of PheDH-AuNCs were recorded after spiking 20-fold diluted human serum with Cys or GSH solutions at final concentrations of 0 to 20 μM under optimized conditions.

### 2.9. *In vitro* cytotoxicity and cellular imaging

The cell line used in this study was obtained from the national cell bank located in the Pasteur institute of Iran. In order to investigate the *in vitro* cytotoxicity of the PheDH-AuNCs, the MTT test was performed on SK-BR-3 cell lines. In brief, 200 μL of SK-BR-3 cells with a density of approximately 2 × 10^4^ cells per well were seeded into a 96-well plate. After incubation (37 °C, humidified 5% CO_2_, 24 h), the culture medium was removed and various concentrations of the PheDH-AuNCs (0.05, 0.1, 0.2, 0.5, 1, 2, 5 mg mL^−1^) were added and the cells were incubated for an additional 24 h. After the 24 hours incubation period, the MTT assay was used to assess the cytotoxicity of the PheDH-AuNCs by measuring the reduction of the formazan product produced by viable cells. After removing the treatment medium, 100 μL of MTT salt solution (0.5 mg mL^−1^ MTT in Dulbecco's modified Eagle's medium (DMEM)) was added to the wells, and incubation was done for 4 hours. Finally, the MTT solution was removed and 100 μL of MTT solubilizing agent (DMSO) was added. The plate was shaken for 20 minutes in the dark, and the absorbance of the formazan product was then measured using a spectrophotometer at a wavelength of 570 nm. The viability of the cells was examined by comparing them with untreated cells as a control.

For fluorescence bioimaging experiments, the 100 × 10^3^ cells per well of SK-BR-3 cells were cultured on 12-well plates (SPL Life Sciences Co., Ltd. Korea) with DMEM culture media containing 10% fetal bovine serum (FBS) and 1% (v/v) penicillin–streptomycin. After overnight incubation at 37 °C and 5% CO_2_, the medium was removed. Afterward, the cells were treated with PheDH-AuNCs for another 12 h at 37 °C and 5% CO_2_. Then, after discarding medium, the cells were washed three times by PBS buffer (pH = 7.4) and finally, the cellular images were captured by a fluorescence microscope equipped with a blue filter.

## 3. Results and discussion

### 3.1. Expression and purification of PheDH enzyme

The expressed histidine-tailed *B. badius* PheDH was purified by Ni-Sepharose affinity chromatography. The results obtained from the SDS-PAGE analysis indicated that the recombinant PheDH with molecular weight of 42 kDa was efficiently purified (Fig. S2†).

### 3.2. Formation, optimization, characterization, and stability of PheDH-AuNCs

**Fig. 1:**
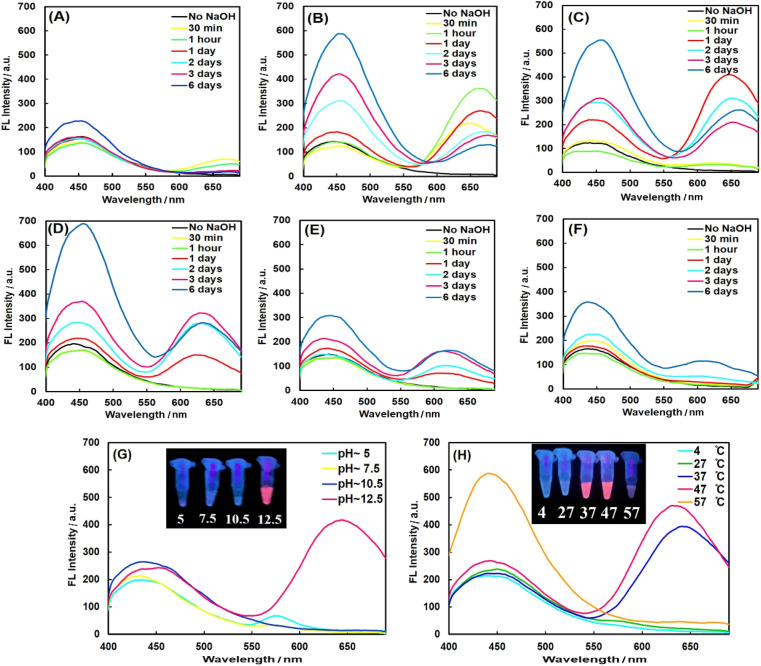
Optimization of synthesis conditions of PheDH-AuNCs. Fluorescence spectra of PheDH-AuNCs at constant concentration of PheDH (4.5 mg mL^−1^) and different concentrations of HAuCl_4_: (A) 0.25, (B) 0.5, (C)1, (D) 1.5, (E) 2 and (F) 3.5 mM at different reaction times (*λ*_ex_ = 360 nm). Effect of (G) pH and (H) temperature on the synthesis of PheDH-AuNCs. The insets show the photographs of PheDH-AuNCs under UV irradiation.

PheDH was used as both capping and reducing agent for one-step green synthesis of fluorescent AuNCs under alkaline conditions. Spectrofluorimetric measurements show two distinct blue and red emission peaks with the single excitation at 360 nm, under optimized conditions, which could be attributed to the formation of two different cluster sizes (blue-emitting and red-emitting clusters).^11^ In order to obtain PheDH-stabilized AuNCs with highest fluorescence intensity, all the key parameters were optimized. The fluorescence measurements upon excitation at 360 nm were used to explore these experiments. The relative amount of the Au precursor to protein was critical in the synthesis of protein-capped AuNCs.^37^ As can be seen from Fig. 1A–F, an aqueous solution containing 1 mM of HAuCl_4_ and ∼4.5 mg mL^−1^ of PheDH in equal volumes with reaction time of 24 h indicated the highest red emission intensity (see Fig. S3† for more detail). After that, the influence of pH value on the synthesis of PheDH-capped AuNCs was investigated. The results indicated that the red fluorescence emission was obtained at a pH value of ∼12.5, whereas reaction solution with initial pH of ∼10.5 showed no red fluorescence emission (Fig. 1G). This indicates that the pH is an important factor in protein conformation and its reducing/capping ability,^28^ so that the pH of the reaction solution could strongly affect the formation of PheDH-AuNCs.

Since this pH value (∼12.5) is higher than the pKa of tyrosine (∼10), the 13 Tyr residues in PheDH can more strongly reduce Au^3+^ ions to Au atoms through the phenolic groups.^37,43,50^ The effect of reaction temperature on the fluorescence intensity of the PheDH-AuNCs was also studied. As is obvious from Fig. 1H, the synthesized AuNCs showed red fluorescence emission when the reactions at 37 and 47 °C were carried out for 24 h (higher red emission occurred at 47 °C). Therefore, 1 mM of HAuCl_4_, ∼4.5 mg mL^−1^ of PheDH, 47 °C and 24 h were chosen as the optimal experiment conditions for the synthesis of PheDH-stabilized AuNCs.

**Fig. 2:**
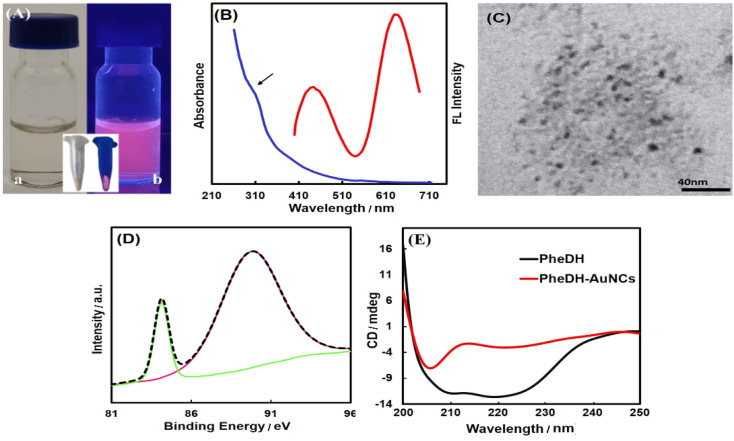
Characterization. (A) Photographs of the PheDH-AuNCs aqueous solutions under (a) visible and (b) UV light; the insets show PheDH-AuNCs in powder form under visible and UV light. (B) Absorption (blue line) and emission spectra (red line, *λ*_ex_ = 360 nm) of PheDH-AuNCs. (C) TEM image of the as-prepared PheDH-AuNCs. (D) XPS spectrum of Au 4f region for PheDH-AuNCs. (E) Far UV-CD spectra of PheDH and PheDH-AuNCs.

As depicted in Fig. 2A, the pale yellow-colored synthesized PheDH-AuNCs exhibit a strong red emission under ultraviolet light. From the absorption spectrum of PheDH-AuNCs solution shown in Fig. 2B, only a weak absorption peak at ∼280 nm is observed, which is attributed to the aromatic amino acid residues of PheDH. Moreover, there is no absorption peak at ∼520 nm corresponding to the surface plasmon resonance (SPR) of gold nanoparticles which indicates the successful synthesis of gold nanoclusters.^43^ In addition, considering the ultra-small size of fluorescent nanoclusters, TEM image was applied to confirm the formation of nanoclusters. As shown, the PheDH-capped AuNCs are approximately spherical with an average size of 3.5 ± 0.7 nm (Fig. 2C). The EDAX results also indicate the presence of Au (Fig. S4†). The synthesis of PheDH-AuNCs was also confirmed by XPS analysis and the results are presented in Fig. 2D. The XPS spectrum of the Au 4f shows a peak at binding energy of 84.1 eV, which is ascribed to 4f_7/2_, confirming the presence of Au(0) valence state.^30,37,43^ The structural changes of PheDH in PheDH-AuNCs system was investigated by CD measurements. Fig. 2E provides the results obtained from the Far-UV CD spectra of free PheDH and PheDH-AuNCs. A comparison of the two graphs reveals that formation of AuNCs causes a conformational change of the PheDH. A significant decrease of enzyme activity after the synthesis of clusters compared to the native enzyme was also observed. Anyway, the experimental conditions for synthesis of nanoclusters such as high pH probably affected the structure and consequently the activity of the PheDH.

**Fig. 3:**
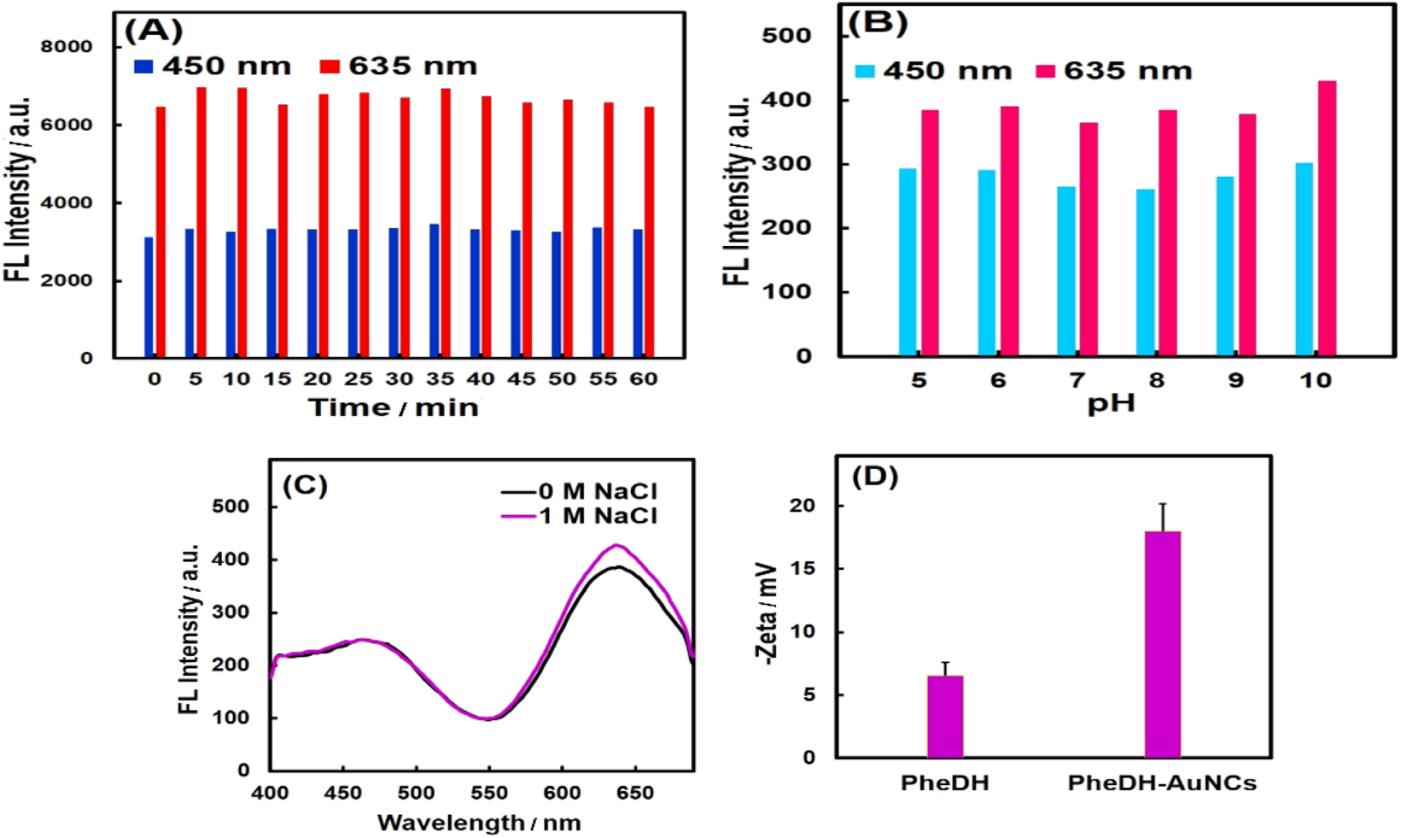
Stability tests. The effect of (A) UV irradiation time and (B) pH on the blue and red emission peaks of PheDH-AuNCs. (C) Fluorescence emission spectra of PheDH-AuNCs in the absence and presence of NaCl (1 M). (D) Zeta potential of PheDH and PheDH-AuNCs under the same conditions.

The stability test results of PheDH-AuNCs have been shown in Fig. 3. As seen, the PheDH-AuNCs presented an excellent photostability (Fig. 3A). As depicted, the emission peaks centered at 450 and 635 nm exhibited no significant change under different UV irradiation times with 5 min intervals from 0 to 60 min (*λ*_ex_ = 360 nm). The effect of different pHs (5–10) on the fluorescence intensity of prepared AuNCs in both blue and red emission peaks was also studied (Fig. 3B). The results revealed that the fluorescence intensity remained almost constant, although the red emission peak increased slightly at alkaline pHs.

The synthesized AuNCs also demonstrated great stability under condition of high ionic concentration (Fig. 3C), because no clear change in fluorescence spectrum was observed for PheDH-AuNCs after adding 1 M NaCl in solution. It seems possible that the good stability of PheDH-AuNCs is due to the size of PheDH (380 aa) and the good coverage of surface atoms and as a result, the inhibition of cluster aggregation.^11^ Furthermore, the good dispersion of PheDH-AuNCs in aqueous solution was confirmed by measuring their zeta potential. The surface charge of PheDH-AuNCs with a zeta potential of −18 mV is more negative than that of PheDH alone (−6 mV) under the same condition that confirms the formation of PheDH-AuNCs and acceptable electrostatic repulsion between particles.^4^ Anyway, at 4 °C, the PheDH-AuNCs in both liquid and powder forms remained stable for at least 1 year without any precipitation or aggregation and preserved their fluorescence. Taken together, these results suggest that PheDH-stabilized AuNCs have great potential for bioimaging, drug delivery and biosensing studies due to their excellent photo and chemical stability, small size, water solubility and low toxicity.

### 3.3. Ratiometric fluorescent detection of Hg^2+^, Cu^2+^, Cys and GSH using PheDH-AuNCs

**Fig. 4:**
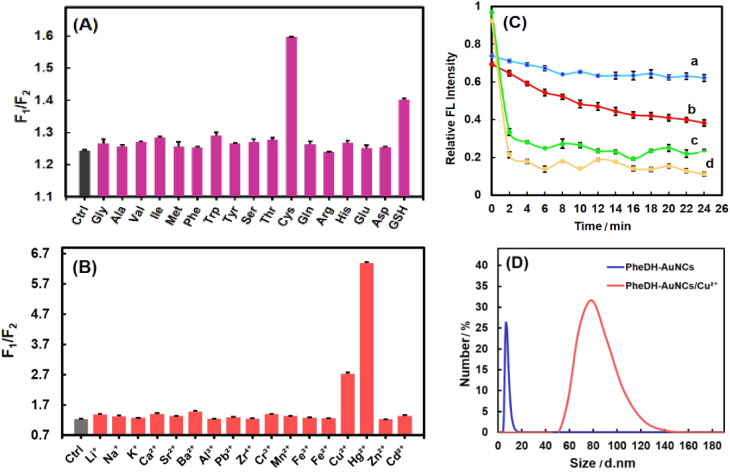
Fluorescence ratio (*F*_1_/*F*_2_) of the PheDH-AuNCs (*λ*_ex_ = 360 nm) in the presence of (A) 100 μM of amino acids and GSH and (B) 12 μM of different metal ions. (C) Time dependent response of the PheDH-AuNCs after addition (a) GSH, (b) Cys, (c) Cu^2+^ and (d) Hg^2+^. (D) Hydrodynamic size measurements of the PheDH-AuNCs in the absence and presence of Cu^2+^ (2 μM) by DLS analysis.

The PheDH-stabilized AuNCs as a great fluorescence probe can be applied for highly sensitive and selective detection of heavy metal ions (Hg^2+^ and Cu^2+^) and important biothiols (Cys and GSH) through ratiometric detection method. At first, the fluorescence intensity of the PheDH-AuNCs was examined in the presence of 16 amino acids and GSH at final concentration of 100 μM. As is obvious from Fig. 4A, the red emission of the PheDH-AuNCs was efficiently quenched by the addition of Cys and GSH and blue emission remained almost constant. On the contrary, other amino acids had no noticeable influence on the fluorescence intensity of PheDH-AuNCs. This could be attributed to the interaction between free SH groups and AuNCs and thus forming an Au–thiol complex as previously reported.^20,53,54^ As well as, the fluorescence response of PheDH-AuNCs was investigated in the presence of various cations at final concentrations of 12 μM. As depicted in Fig. 4B, Hg^2+^ and Cu^2+^ ions result in a significant quenching of the red emission of PheDH-AuNCs, while the other metal ions produce negligible effects on the emission of PheDH-AuNCs. Several studies revealed the possible mechanisms of fluorescence quenching of AuNCs in the presence of these ions. The quenching of the red emission peak of PheDH-AuNCs in the presence of Cu^2+^ ions can be mainly attributed to the aggregation of gold nanoclusters caused by the interaction between histidyl and carboxyl groups of PheDH shell of the clusters and Cu^2+^ ions.^4,15,51^ Moreover, the luminescence quenching of AuNCs in the presence of Hg^2+^ is ascribed to the strong specific metallophilic interaction between Au^+^ and Hg^2+^ ^9,19,23^ that could be resulted in cluster aggregation^15,52^ The aggregation-caused quenching (ACQ) process could be confirmed by DLS analysis. Among the introduced targets, DLS measurements were performed before and after the addition of Cu^2+^ as a model. As can be seen, the hydrodynamic diameter of PheDH-AuNCs was increased in the presence of Cu^2+^ (Fig. 4D).

The response time of the PheDH-AuNCs to Cys, GSH, Hg^2+^ and Cu^2+^ were investigated over different times. It was found that the fluorescence quenching of the PheDH-AuNCs did not exhibit dramatic changes with elapse of time in the presence of Hg^2+^ and Cu^2+^; but the red emission of the PheDH-AuNCs was significantly quenched in the presence of Cys and GSH within about 10 and 5 min, respectively, and then approached a constant value (Fig. 4C). So, for subsequent experiments, we selected 10 and 5 min as appropriate detection times for Cys and GSH, respectively. Generally, the results of selectivity studies clearly indicate that PheDH-AuNCs can be used as a nano-biosensor for highly selective detection of Hg^2+^, Cu^2+^, Cys and GSH *via* ratiometric fluorescence mechanism.

**Fig. 5:**
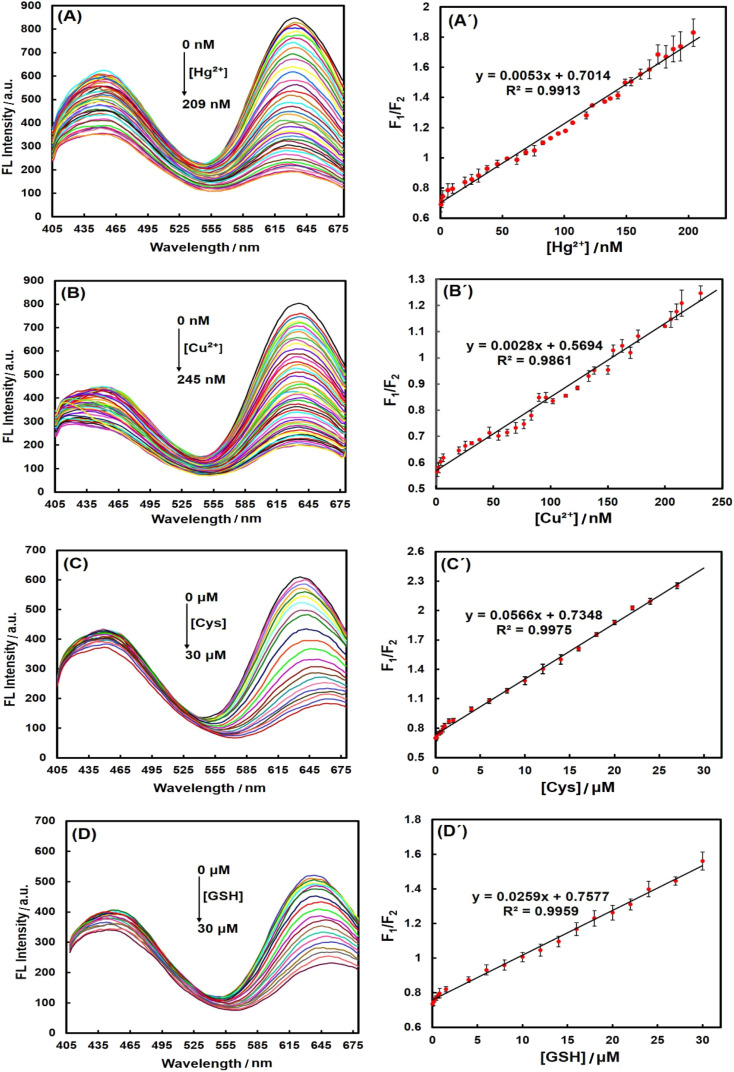
Fluorescence emission response of PheDH-AuNCs (*λ*_ex_ = 360 nm) upon addition of different concentrations of (A) Hg^2+^, (B) Cu^2+^, (C) Cys and (D) GSH. Calibration graphs of fluorescence intensity ratio (*F*_1_/*F*_2_) of PheDH-AuNCs *versus* (A’) Hg^2+^, (B’) Cu^2+^, (C’) Cys and (D’) GSH concentration. ‘‘*F*_1_” and ‘‘*F*_2_” represent the blue and red emission peaks, respectively.

In the next step, a series of calibration experiments were performed separately using various concentrations of analytes (from 0 to 209 nM for Hg^2+^, 0 to 245 nM for Cu^2+^ and 0 to 30 μM for Cys and GSH) to assess the range of linear detection. The spectra in Fig. 5 demonstrated that the red fluorescence intensity of PheDH-AuNCs was gradually decreased with increasing the concentration of Hg^2+^, Cu^2+^, Cys and GSH, separately, while the blue emission peak exhibited only a slight change. The corresponding plots of the emission intensity ratio (*F*_1_/*F*_2_: where ‘‘*F*_1_” represents the blue emission peak and ‘‘*F*_2_” displays the red emission peak) *versus* Hg^2+^, Cu^2+^, Cys and GSH concentrations showed excellent linear correlations with correlation coefficients (*R*^2^) of 0.9913, 0.9861, 0.9975, 0.9959, respectively (Fig. 5). To demonstrate reproducibility, the standard deviation was calculated for the results, and the length of the error bars in the graphs represent the standard deviation. The limit of detection (LOD) was then calculated based on the equation of 3 SD/*m*^55,56^ (where SD is the standard deviation for 3 replicates in blanks, and *m* is the slope of the corresponding linear equation).

The LODs were obtained 1.6 and 2.4 nM for Hg^2+^ and Cu^2+^ ions, respectively, which are much lower than the maximum permissible levels of these heavy metal ions in drinking water (0.002 ppm ∼10 nM for Hg^2+^ and 1.3 ppm ∼20 μM for Cu^2+^) established by the environmental protection agency (EPA) of the United States.^28^ The LOD for Cys was obtained 0.16 μM that is lower than that of GSH (0.35 μM), because from the data in Fig. 4A and C, it is apparent that PheDH-AuNCs are more sensitive to Cys compared to GSH. Typically, the normal cysteine concentration in human blood plasma and cells is about 240–360 μM and 30–200 μM, respectively.^54,57,58^ Normal level of GSH in blood plasma is in the micromolar range and in the cytosol of cells is about 1–10 mM.^59,60^ However, the LODs obtained for Cys and GSH in the present study are lower than their normal ranges in adult human plasma and cells. In Table 1, the detection method, linear dynamic range (LDR) and LOD of some previous reports for the detection of Hg^2+^, Cu^2+^, Cys and GSH using AuNCs are compared with those obtained in this study. It is apparent that our LOD values are lower than or comparable to most previously reported detection methods. These results confirm that the ratiometric PheDH-AuNC probe possess great sensitivity for selective detection of Hg^2+^, Cu^2+^, Cys and GSH, so the PheDH-AuNCs can be effectively used for the detection of these analytes. Regarding to further improve the selectivity of this developed sensor toward one target, some extra reactions could be used to discriminate the source of the quenching. For example, it has been reported that Cu^2+^-induced fluorescence quenching can be recovered in the presence of Cu^2+^ chelator such as EDTA,^46^ His^4,46^ or Gly^61^ and Hg^2+^-induced AuNCs quenching can be restored by using sodium borohydride, because NABH_4_ with reduction Hg^2+^ into Hg^0^, lead to much weaker binding Hg^2+^ to Au^+^.^46,62^ Furthermore, the effect of Cys and GSH can be selectively eliminated by using *N*-ethylmaleimide (NEM) as a thiol-masking agent.^14,63,64^

**Table 1:** Comparison of PheDH-AuNCs with some previously reported protein-capped AuNCs for the detection of Hg^2+^, Cu^2+^, Cys and GSH

Probe	Method	Linear range	LOD (nM)	Ref.
**Fluorescence probes for determination of Cu** ^**2+**^				
BSA-AuNCs	Signal off	30–500 μM	500	65
BSA-AuNCs	Signal off	0.5–100 μM	300	46
Papain-AuNCs	Signal off	10–250 nM	3	39
Hemoglobin-AuNCs	Signal off	0.1–20 μM	28	4
BSA-AuNCs	On-off switch	0.5–30 μM	146.5	22
BSA-AuNCs- La^3+^	Signal off	0.05–10 μM	48	15
PheDH-AuNCs	Ratiometric	0–231 nM	2.4	This work

**Fluorescence probes for determination of Hg** ^**2+**^				
BSA-AuNCs	Signal off	0.4–43.2 μM	80	66
BSA-AuNCs	Signal off	1–20 nM	0.5	62
Pepsin-AuNCs	Signal off	1–200 nM	1	67
Trypsin-AuNCs	Signal off	50–600 nM	∼50	68
BSA-AuNCs	Signal off	10–250 nM	4	19
BSA-AuNCs	Signal off	25–5000 nM	8	46
Fibrinogen-AuNCs	Signal off	0.01–10 μM	150	14
BSA-AuNCs-La^3+^	Signal off	0.05–15 μM	20	15
PheDH-AuNCs	Ratiometric	0–209 nM	1.6	This work

**Fluorescence probes for determination of Cys**				
BSA fibril-AuNCs	Signal off	0.076–300 μM	76	54
Fibrinogen-AuNCs	Signal off	0.01–150 μM	790	14
BSA-AuNCs-NBD	Ratiometric	8.33–100 μM	1450	56
PheDH-AuNCs	Ratiometric	0–30 μM	160	This work

**Fluorescence probes for determination of GSH**				
BSA-AuNCs	Off–on switch	0.04–16.0 μM	7	47
Transferrin-AuNCs	Off–on switch	0–150 μM	2860	21
PheDH-AuNCs	Ratiometric	0–30 μM	350	This work

a Signal off: fluorescence quenching, signal on: fluorescence enhancement, off–on switch: fluorescence recovery, on–off switch: enhanced fluorescence quenching, NBD: 7-nitro-2,1,3-benzoxadiazole.

Recovery tests were performed to investigate possible matrix effects and method accuracy by spiking real samples with different concentrations of each analyte, independently. The results have been presented in Table 2.

Results of recovery tests from tap and mineral water spiked with Hg^2+^ and Cu^2+^ and from human blood serum spiked with Cys and GSH by PheDH-AuNCs^a^

**Table:** 

Sample	Spiked (nM)	Found (nM) ± SD	Recovery (%) ± SD	Found (nM) ± SD	Recovery (%) ± SD	ICP (nM)
Mineral water		Hg^2+^		Cu^2+^		
	0	3.6 ± 0.3	—	3.4 ± 0.08	—	Hg^2+^: <249.2
						Cu^2+^: <314.7
	10	15.6 ± 0.9	120 ± 7	15.1 ± 1.5	116.7 ± 14	
	20	24 ± 0.9	101.8 ± 4.9	27.5 ± 0.8	120 ± 5.6	
	40	43 ± 1.6	98.3 ± 4.9	43.7 ± 1.6	100.7 ± 6.1	
	80	81.5 ± 0.1	97.3 ± 0.3	82.8 ± 0.2	99.2 ± 0.4	
	100	105 ± 0.4	101.3 ± 1	103.7 ± 0.5	100.2 ± 0.4	
Tap water	0	2.8 ± 0.1	—	6.1 ± 0.06	—	Hg^2+^: <249.2
						Cu^2+^: <314.7
	10	14.5 ± 0.5	117.3 ± 6.8	18 ± 0.9	119 ± 10	
	20	23.6 ± 1.8	104.2 ± 8	29.3 ± 1.1	115 ± 8.7	
	40	43.8 ± 2	102.5 ± 7.7	47.7 ± 0.9	104 ± 3.3	
	80	81.2 ± 2	98 ± 4.7	84.3 ± 0.7	97.7 ± 1.3	
	100	105 ± 0.2	102.7 ± 0.2	106.5 ± 1.1	100.4 ± 1.7	

a SD: standard deviation.

**Table:** 

Sample	Spiked (μM)	Found (μM) ± SD	Recovery (%) ± SD	Found (μM) ± SD	Recovery (%) ± SD
Human serum		Cys		GSH	
	0	Not detected	—	Not detected	—
	2.5	2.42 ± 0.07	96.8 ± 4	2.82 ± 0.004	113 ± 0.2
	5	5.7 ± 0.05	114 ± 1	5.23 ± 0.1	104.6 ± 4.8
	10	10.5 ± 0.001	105 ± 0.02	9.4 ± 0.5	93.7 ± 7
	20	19.6 ± 0.009	98 ± 0.07	20.2 ± 0.1	101 ± 1.1

As can be seen, the recovery values using the developed nanobiosensor were found to be between 97.3% and 120% for heavy metal ions and also between 93.7% and 114% for biomarker thiols. These resulting good recoveries exhibit the satisfactory accuracy and high reliability of our designed method, which can be used for the detection of these analytes in real samples.

### 3.4. Cytotoxicity and bioimaging

**Fig. 6:**
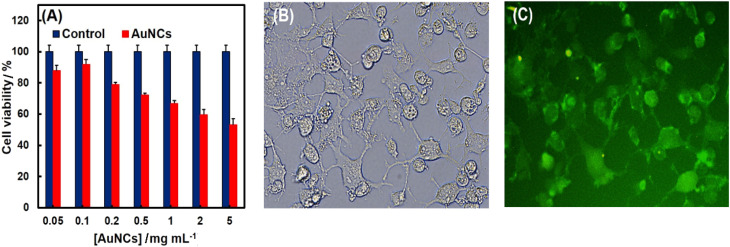
*In vitro* cytotoxicity of PheDH-AuNCs and cellular imaging. (A) MTT test after 24 h of treatment against SK-BR-3 cells. (B) Bright field and (C) fluorescence imaging of SK-BR-3 cells co-incubation with PheDH-AuNCs (2 mg mL^−1^) for 12 h.

The cell viability and *in vitro* cytotoxicity were evaluated using SK-BR-3 cells through the MTT assay method. As shown in Fig. 6A, results demonstrated a dose-dependent cytotoxicity of PheDH-AuNCs and good cell viability from 0.05 to 1 mg mL^−1^ (∼70% after 24 h incubation). Thus, the PheDH-AuNCs exhibited a bio-compatible nature and can be used as a potential candidate for *in vitro* imaging. Thus, the applicability of the PheDH-AuNCs then checked in live-cell imaging. As revealed by the fluorescence microscopy (Fig. 6B and C), the intense fluorescence of PheDH-AuNCs was detected in the SK-BR-3 cells and AuNCs were found in the whole cell region. Based on these findings, the PheDH-AuNCs indicated great cell permeability and promising opportunities for cellular imaging and bio-medical applications.

## 4. Conclusions

In this work, for the first time, PheDH enzyme as a new great template is proposed for the simple, one-pot and very friendly synthesis of the multifunctional label-free fluorescent AuNCs. The prepared PheDH-capped gold nanoclusters (PheDH-AuNCs) exhibit dual fluorescent emission with single excitation and interestingly remain stable for more than a year. We have found that only the red emission of PheDH-AuNCs can be effectively quenched in the presence of mercury, copper, cysteine and glutathione. Therefore, the PheDH-AuNCs were utilized as an excellent ratiometric fluorescent probe to develop fast, label-free, surface modification-free, sophisticated equipment-free, low cost and highly sensitive and selective bioassay system for the detection of heavy metal ions and biomarker thiols with wide linear ranges and a lower LOD compared to most previous reports. In addition, the cytotoxicity and cell imaging studies clearly demonstrated that the PheDH-AuNCs as a novel biocompatible optical probe can be applied for fluorescence bioimaging and biological or medical applications.

## Author contributions

Mahsa Shahrashoob: methodology, investigation, formal analysis, writing – original draft & editing. Saman Hosseinkhani: supervision, validation, funding acquisition, review & editing. Hanieh Jafary: review & editing. Morteza Hosseini: resources, validation. Fatemeh Molaabasi: validation, project administration, review & editing.

## Informed consent

Informed consent was obtained from all subjects.

## Conflicts of interest

There are no conflicts to declare.

## Supplementary Material

RA-013-D3RA03179A-s001
