# Supplementary material for: Dual-emissive phenylalanine dehydrogenase-templated gold nanoclusters as a new highly sensitive label-free ratiometric fluorescent probe: heavy metal ions and thiols measurement with live-cell imaging
Source: RSC Adv. 2023 Jul 19;13(31):21655–66. doi: 10.1039/d3ra03179a (PMC10354591; doi:10.1039/d3ra03179a)
Supplement: RA-013-D3RA03179A-s001 [file RA-013-D3RA03179A-s001.pdf]

Electronic Supplementary Material (ESI) for RSC Advances.
This journal is © The Royal Society of Chemistry 2023

Electronic Supplementary Information

Dual-Emissive Phenylalanine Dehydrogenase-Templated Gold Nanoclusters as a New Highly Sensitive Label-Free Ratiometric Fluorescent Probe: Heavy Metal Ions and Thiols measurement with Live-Cell Imaging

Mahsa Shahrashoob[a], Saman Hosseinkhani*[b], Hanieh Jafary[a], Morteza Hosseini[c], Fatemeh Molaabasi[d]

[a] Department of Biology, Science and Research Branch, Islamic Azad University, Tehran, Iran

[b] Department of Biochemistry, Faculty of Biological Sciences, Tarbiat Modares University, Tehran, Iran

[c] Department of Life Science Engineering, Faculty of New Sciences & Technologies, University of Tehran, Tehran, Iran

[d] Department of Interdisciplinary Technologies, Breast Cancer Research Center, Biomaterials and Tissue Engineering Research Group, Motamed Cancer Institute, ACECR, Tehran, Iran

* Corresponding Author: Professor Saman Hosseinkhani

E-mail: saman_h@modares.ac.ir

Expression and purification of Phenylalanine dehydrogenase enzyme

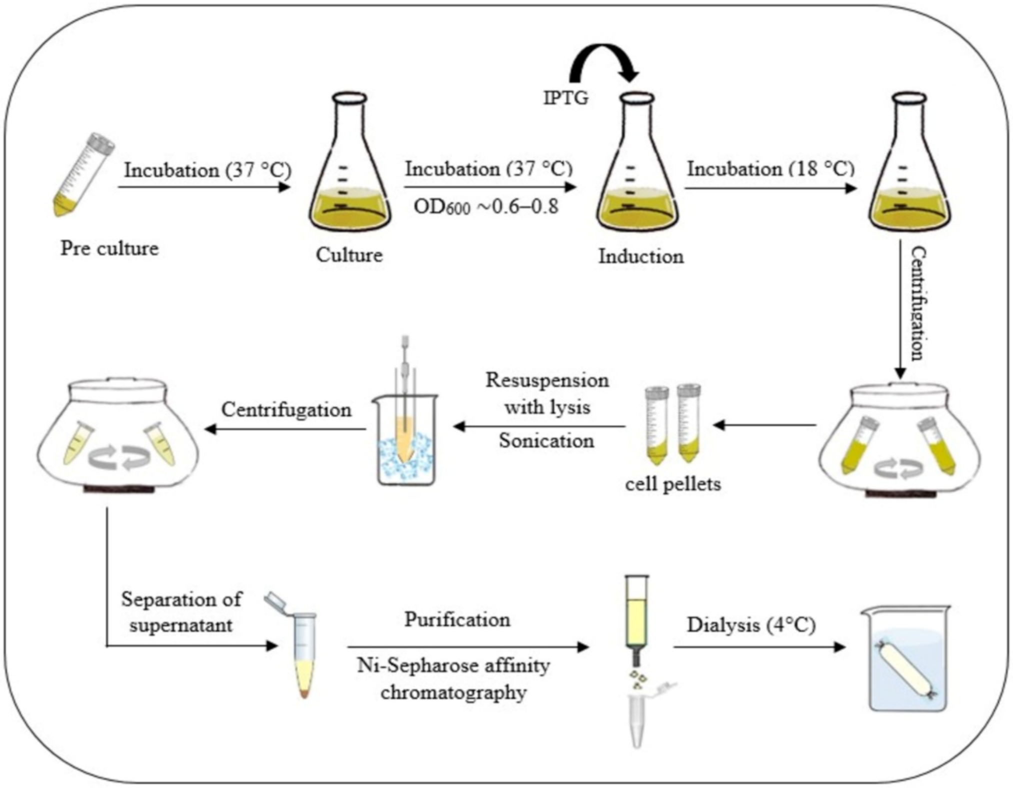

Figure S1. Schematic illustration of the expression and purification steps of the recombinant histidine-tailed PheDH.

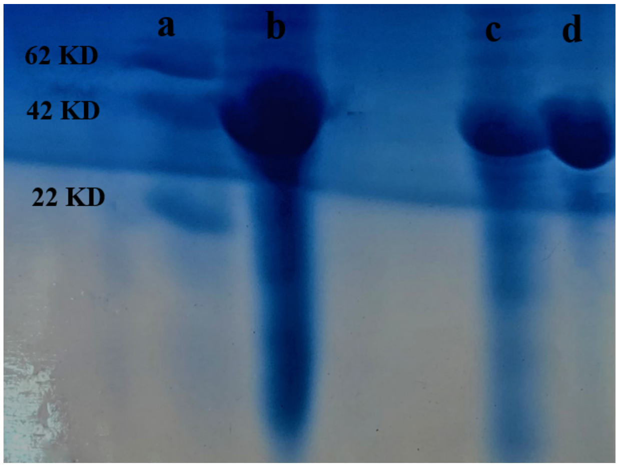

Figure S2. SDS-PAGE analysis of *B. badius* PheDH. (a) Protein marker, (b) bacterial flow soup, (c) washing step and (4) final eluted fractions.

PheDH–capped AuNCs: Optimization and Characterization of the PheDH-AuNCs

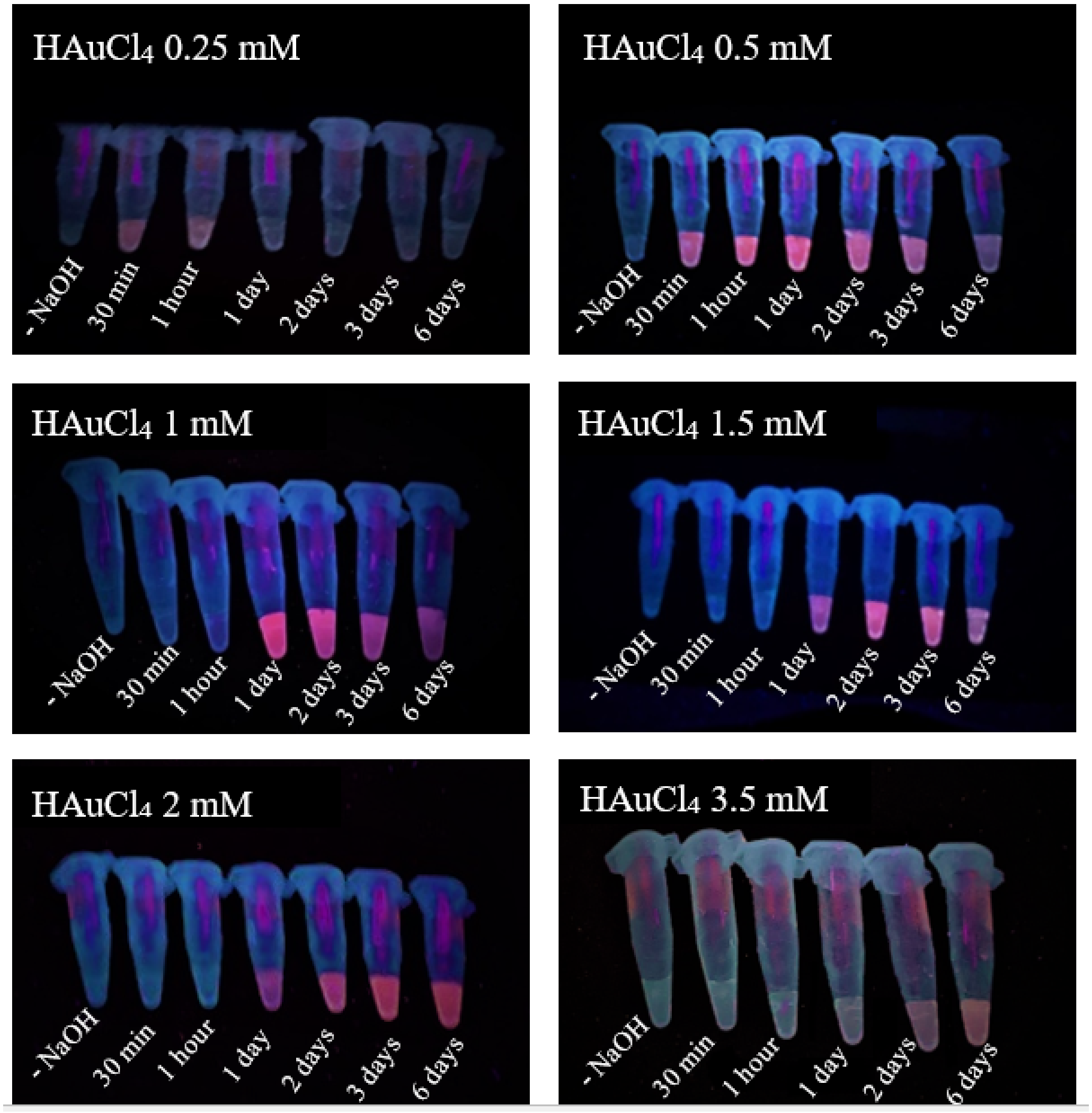

Figure S3. Photographs of the PheDH-AuNCs aqueous solutions at constant concentration of PheDH (4.5 mg ml^{-1}) and different concentrations of $HAuCl_4$ at different reaction times under UV light.

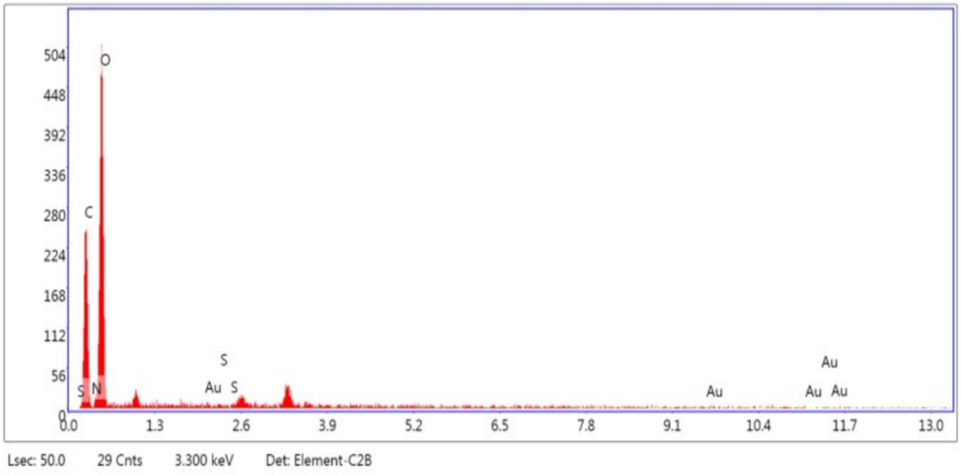

Figure S4. EDAX spectra of the PheDH-AuNCs.

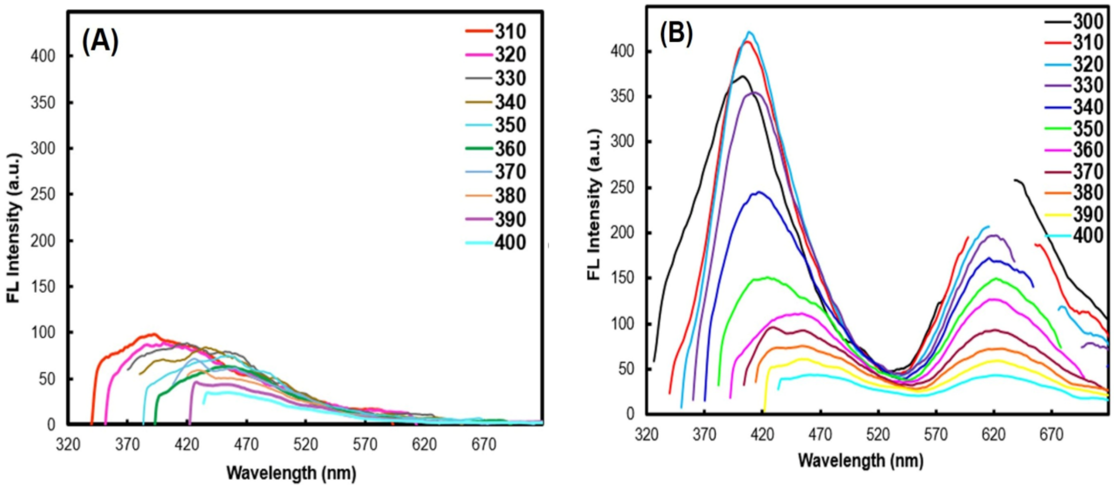

Figure S5. Fluorescence spectra of (A) PheDH enzyme and (B) PheDH-AuNCs recorded at different excitation wavelengths.
